# The simultaneous enhancement of photorefraction and optical damage resistance in MgO and Bi_2_O_3_ co-doped LiNbO_3_ crystals

**DOI:** 10.1038/srep20308

**Published:** 2016-02-03

**Authors:** Dahuai Zheng, Yongfa Kong, Shiguo Liu, Muling Chen, Shaolin Chen, Ling Zhang, Romano Rupp, Jingjun Xu

**Affiliations:** 1 School of Physics, Nankai University, Tianjin 300071, China; 2 MOE Key Laboratory of Weak-Light Nonlinear Photonics and TEDA Applied Physics School, Nankai University, Tianjin 300457, China; 3 R&D Center, Taishan Sports Industry Group, Leling 253600, China; 4 Collaborative Innovation Center of Chemical Science and Engineering (Tianjin), Tianjin 300072, China; 5 Vienna University, Faculty of Physics, A-1090 Wien, Austria

## Abstract

For a long time that optical damage was renamed as photorefraction, here we find that the optical damage resistance and photorefraction can be simultaneously enhanced in MgO and Bi_2_O_3_ co-doped LiNbO_3_ (LN:Bi,Mg). The photorefractive response time of LN:Bi,Mg was shortened to 170 ms while the photorefractive sensitivity reached up to 21 cm^2^/J. Meanwhile, LN:Bi,Mg crystals could withstand a light intensity higher than 10^6^  W/cm^2^ without apparent optical damage. Our experimental results indicate that photorefraction doesn’t equal to optical damage. The underground mechanism was analyzed and attributed to that diffusion dominates the transport process of charge carriers, that is to say photorefraction causes only slight optical damage under diffusion mechanism, which is very important for the practical applications of photorefractive crystals, such as in holographic storage, integrated optics and 3D display.

Optically-induced refractive index inhomogeneities were firstly observed in LiNbO_3_ (LN) and LiTaO_3_^1^. This effect, although interesting in its own right, poses serious limitations in the use of these crystals in nonlinear devices requiring high light intensity, such as frequency doubler, optical parametric oscillator, Q-switcher, and integrated optics^2^,^3^,^4^,^5^,^6^, so later was called as optical damage. However, this same effect can be used to advantage to form a holographic recording in the applications where a material gives refractive index change directly upon exposure, so it was renamed as photorefraction (PR), and LN was found as an extremely interesting holographic media^7^. From then on, many works were conducted to control PR or optical damage. And dopants were found having extremely influence on the PR of LN, such as Fe, Cu, Mn, Ni and Ce^8^,^9^,^10^ can greatly enhance PR, on the other hand, optical damage can be greatly degraded by Mg, Zn, In and Sc^11^,^12^,^13^,^14^ and recently reported Hf, Zr and Sn^15^,^16^,^17^. Till now, photorefraction enhancement and optical damage resistance are two main research directions of LN crystals on nonlinear optics or photonics. However, in this paper we found that optical damage resistance and PR of LN can be enhanced simultaneously by Bi and Mg co-doping. This phenomenon implies that the mechanism of optical damage and the relationship between photorefraction and optical damage should be reconsidered.

The electron lone-pair of Bi^3+^ ion (6*s*^2^) always induces high polarizability in crystal lattice, which causes many interests in ferroelectrics, nonlinear optics and electro-optical properties. In a former work^18^, we reported the photorefractive characteristics of Bi doped LiNbO_3_ crystals. It was shown that bismuth dopants may introduce new photorefractive centers to LN crystals. On the other hand, Mg doped LN crystals are famous for its high resistance to optical damage^11^, and Mg and Fe or Mo co-doped LN crystals show greatly enhanced photorefractive properties^19^,^20^. Here Bi and Mg were co-doped into LN (LN:Bi,Mg) crystals and their photorefractive properties, optical damage resistance, Uv-visible absorption spectra and OH^−^ absorption spectra were investigated. It was found that LN:Bi,Mg crystals have simultaneously enhanced PR with high optical damage resistance, and the mechanism about this phenomenon was discussed.

## Results and Discussion

### The photorefractive properties

The photorefractive properties of LN:Bi,Mg crystals were investigated by two-wave coupling method in transmission geometry. The laser beam polarization vector lied in the plane of beam incidence to exploit the largest electro-optic coefficient r_33_. Two wavelengths, 532 nm from a cw frequency-doubled solid-state laser and 488 nm from an Ar laser, were chosen for the measurements. The extraordinary polarized laser was split into two beams with equal light intensity (per beam 400 mW/cm^2^). The two beams transmitted the same optical path, and illuminated the 3.0 mm y-oriented plates with a crossing angle 30°. The diffraction efficiency is defined as *η* = *I*_d_/(*I*_d_ + *I*_t_), where *I*_d_ and *I*_t_ is the diffracted and transmitted light intensity of the readout beam, respectively. The photorefractive response time constant *t*_r_ and the saturated diffraction efficiency *η*_s_ are described by the function of 
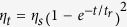
. The change of the refractive index Δn is calculated from *η* = sin^2^*(πd*Δ*n/λ*cos*θ)*, where *λ* is the free-space wavelength, *d* is the length of grating within the sample, and *θ* is the Bragg angle. The photorefractive sensitivity is defined as 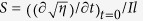, where *I* is the total recording light intensity and *l* is the thickness of crystal plates. For detail experimental setups and parameter definition, one can see ref. 21.

**Figure 1:**
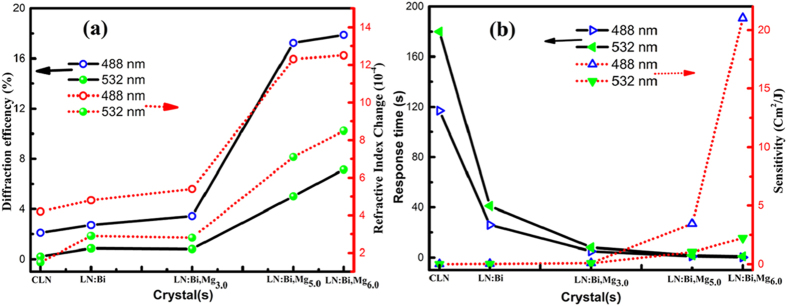
(**a**) The diffraction efficiency (left) and refractive index change (right) and (**b**) The photorefractive response time (left) and sensitivity (right) of LN:Bi,Mg crystals as functions of the doping concentration of Mg. For comparison, the data of nominally pure congruent LN(CLN) and 1.0 mol% Bi mono-doped LN (LN:Bi) crystals were also drawn.

The diffraction efficiency, refractive index change, photorefractive response time, and sensitivity of LN:Bi,Mg crystals are shown in Fig. 1. For comparison, the data of nominally pure congruent LN (CLN) and 1.0 mol% Bi mono-doped LN (LN:Bi) crystals were also drawn in this figure. We can see from Fig. 1(a) that the diffraction efficiency and refractive index change increase with increased doping concentration of Mg (C_Mg_), and the diffraction efficiency of LN:Bi,Mg_5.0_ and LN:Bi,Mg_6.0_ (naming information is shown in Methods section) can reach 17.2% and 18.0% in 488 nm laser, respectively. The photorefractive response time of LN:Bi,Mg crystals are shown in Fig. 1(b), which are significantly shorter than that of CLN and LN:Bi. Especially, the response time of LN:Bi,Mg_6.0_ is below 170 ms at 488 nm. The photorefractive sensitivity of LN:Bi,Mg crystals are also shown in Fig. 1(b). We can see that the photorefractive sensitivity of LN:Bi,Mg crystals are greatly enhanced comparing with that of CLN and LN:Bi, especially the sensitivity of LN:Bi,Mg_6.0_ reaches 21 cm^2^/J, which is three orders of magnitude higher than that of CLN and LN:Bi.

The above experiment results illustrated that the photorefractive performance of LN crystals can be greatly enhanced by Mg and Bi co-doping. Compared with the famous iron doped LN (LN:Fe)^22^, the response time of LN:Bi,Mg is more than two orders of magnitude shorter while the diffraction efficiency is only several times lower, so the photorefractive sensitivity was greatly enhanced. These results indicate that LN:Bi,Mg crystals are more suitable for the practical applications in holographic processing.

### Optical damage resistance ability

Laser-induced optical damage can be measured by the transmitted beam spot distortion method. A transmitted light beam will become smeared and elongated along the c axis and furthermore a decrease of the intensity in the central part. Therefore, the ability of LN:Bi,Mg crystals to resist optical damage can be characterized by the light intensity (before incident), named damage threshold, denoting an onset of distortion of the transmitted laser beam passing through the 3.0 mm y-cut plates.

**Figure 2:**
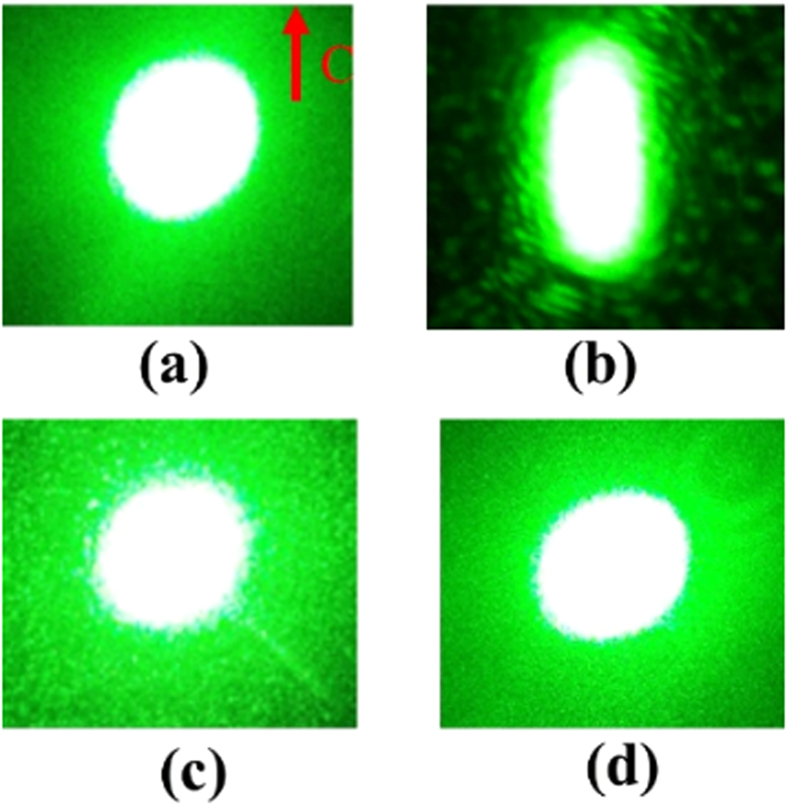
The incident and transmitted laser beam spots after 5 minutes of continuous laser irradiation. (**a**) The incident beam spot, and the transmitted beam spot with (**b**) LN:Bi,Mg_3.0_ crystal, (**c**) LN:Bi,Mg_5.0_ crystal and (**d**) LN:Bi,Mg_6.0_ crystal, respectively, while the light intensity of (**a**,**c,d)** is 5.8 × 10^6 ^W/cm^2^, (**b**) is 7.8 × 10^2 ^W/cm^2^.

The incident and transmitted beam spots with the LN:Bi,Mg crystals after 5 minutes continuous 532 nm laser irradiation are shown in Fig. 2. As shown in Fig. 2(b), the beam spot propagating through LN:Bi,Mg_3.0_ plate distorts along c-axis at a low light intensity of 7.8 × 10^2 ^ W/cm^2^, whereas that of LN:Bi,Mg_5.0_ and LN:Bi,Mg_6.0_ are not apparent smeared compared with the incident beam spot, although the light intensity reaches 5.8 × 10^6 ^W/cm^2^, which is the highest one now we can get in our lab, just as shown in Fig. 2(a,c,d).

As we known, LiNbO_3_ is a typical non-stoichiometric crystal, there are about 1.0 mol% Nb^5+^ ions occupying Li-sites (Nb_Li_^5+^) and 4.0 mol% Li-vacancies (V_Li_) in CLN crystals. A Nb_Li_^5+^ ion captures an electron will form a small polaron (Nb_Li_^4+^) and a Nb_Li_^5+^ ion and a normal Nb^5+^ ion in Nb-site (Nb_Nb_^5+^) capture two electrons will form a bipolaron (Nb_Li_^4+^:Nb_Nb_^4+^), small polaron and bipolaron can transfer each other under suitable light or temperature, they and some valance-changeable impurities (for example Fe^2+/3+^, Cu^+/2+^ and Mn^2+/3+^) act as the photorefractive centers of CLN. When the doping concentration of Mg reaches 4.6 mol% (named as the doping threshold), the optical damage resistance of LN:Mg is at least two orders of magnitude higher than that of CLN^11^. The micro-mechanism of this phenomenon was considered as that Mg^2+^ dopants push Nb_Li_^5+^ ions and photorefractive impurities from Li-sites to Nb-sites, which will cause these ions lose their function as photorefractive centers, therefore the optical damage resistance of LN:Mg is greatly improved. It was reported that the damage threshold of CLN and LN:Bi_1.0_ is lower than 40 W/cm^2^ and that of LN:Mg_5.0_ is around 5 × 10^5 ^W/cm^2^ under the same experimental conditions of this work^17^,^18^. Figure 2 shows that the optical damage threshold of LN:Bi,Mg_5.0_ and LN:Bi,Mg_6.0_ both reach 5.8 × 10^6 ^W/cm^2^. So our experimental results indicate that Bi and Mg co-doping can significantly enhance the optical damage resistance ability of LN crystals. Even compared with LN:Mg, the optical damage resistance of LN:Bi,Mg is further improved one order of magnitude by Bi co-doping. We can see from Fig. 1(b) that the response time of LN:Bi,Mg_5.0_ and LN:Bi,Mg_6.0_ was greatly shortened, in fact, which is at least one order of magnitude shorter than that of LN:Fe,Mg crystal^10^. It was considered that the fast charge transport will reduce the light-induced space charge field and then photorefraction. With strong optical damage resistance, LN:Bi,Mg crystals can be used in high light intensity fields, such as optical parametric oscillator (OPO), second harmonic generation (SHG), and integrated optics.

### The UV-vis absorption spectrum

**Figure 3:**
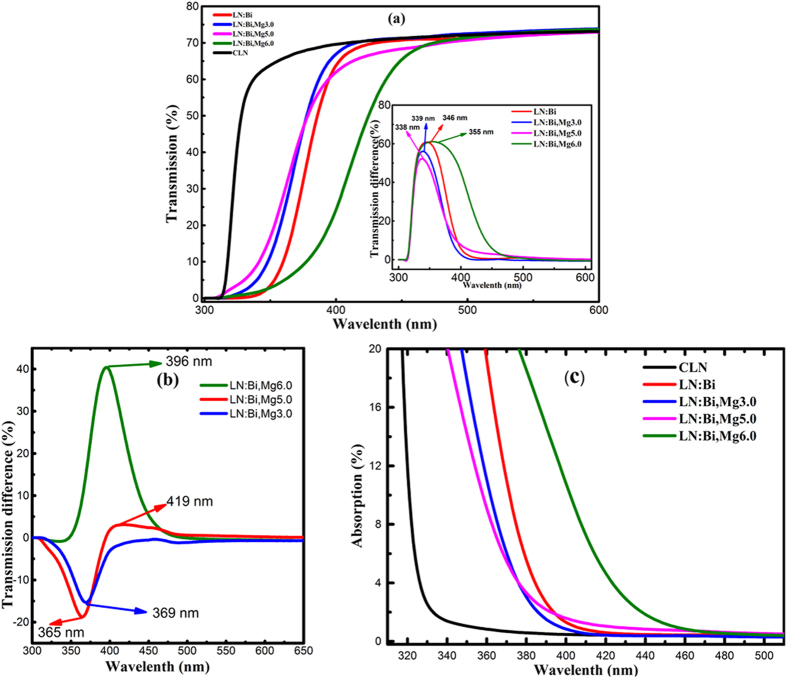
(**a**) The UV-visible transmission spectra of LN:Bi,Mg, and the inset shows transmission difference between LN:Bi,Mg and CLN, which is obtained by subtracting the measured transmission of LN:Bi,Mg from that of CLN. (**b**) The difference transmission spectra of LN:Bi,Mg with that of LN:Bi. (**c**) The UV-visible absorption edges of LN:Bi,Mg crystals. For comparison, the transmission and absorption spectra of CLN and LN:Bi were also drawn.

The absorption spectrum of LN is sensitive to crystal composition and defects, and as we known that Bi dopants can introduced new absorption peaks into LN crystals^18^. The UV-visible transmission spectra of LN:Bi,Mg crystals are shown in Fig. 3(a), we can see that there is obvious absorption at around 350 nm as compared with that of CLN. The inset figure is the transmission spectra difference between LN:Bi,Mg and CLN crystals, it was obtained by subtracting the transmission of LN:Bi,Mg from that of CLN. The peak position of LN:Bi,Mg_5.0_, LN:Bi,Mg_3.0_, LN:Bi and LN:Bi,Mg_6.0_ is 338 nm, 339 nm, 346 nm and 355 nm, respectively. In addition, the peak shapes are different, where LN:Bi,Mg_6.0_ has the widest absorption region. The difference UV-visible transmission spectra between LN:Bi,Mg and LN:Bi are shown in Fig. 3(b), it is interested that there is a transmission peak (369 nm) for LN:Bi,Mg_3.0_, an absorption peak (396 nm) for LN:Bi,Mg_6.0_, and a transmission peak and an absorption peak at 365 nm and 415 nm respectively for LN:Bi,Mg_5.0_. The absorption edge of LN crystal is generally defined as the wavelength where the absorption coefficient equals to 20 cm^−1^. From Fig. 3(c) we can see that the absorption edge of LN:Bi and LN:Bi,Mg are strongly red-shift compared with that of CLN, although the absorption edges of LN:Bi,Mg_3.0_ and LN:Bi,Mg_5.0_ are slight violet-shift in comparison with LN:Bi while that of LN:Bi,Mg_6.0_ is greatly red-shift.

The above results indicate that Bi and Mg co-doping also introduces new absorption centers into LN crystals, which may act as photorefractive centers to influence the photorefractive effects, and the dopants intend to occupy different positions depending on the doping concentration. We can see from Fig. 3(a,c) that the absorption peak and edge of LN:Bi,Mg shift at first to violet side with increased doping concentration of Mg and then to red side as the doping concentration of Mg above its threshold. These phenomena are similar with that of Mg mono-doped LN. It was considered that Mg^2+^ ions will occupy Li-sites and push anti-site Nb_Li_^5+^ ions to normal Nb-sites when the concentration of Mg is below the doping threshold, because the valance of Mg^2+^ is near that of Li^+^ and the defect cluster of Mg_Li_^2+^ − V_Li_ induce slighter lattice distortion as compared with Nb_Li_^5+^ − 4V_Li_, this process will cause a more perfect lattice, a wider band gap and a violet-shift absorption. When the concentration of Mg is above the doping threshold, there is no Nb_Li_^5+^ ion to be substituted, Mg^2+^ ions will occupy Nb-sites, and the defect cluster of 3Mg_Li_^2+^ − Mg_Nb_^2+^ will induce large lattice distortion and red-shift absorption. Because the doping concentration of Bi is much smaller than that of Mg in our samples, the absorption edge of LN:Bi,Mg is mainly depending on the doping concentration of Mg and the absorption peak corresponding to Bi ions is also strongly affected by Mg dopants.

### The OH^−^ absorption spectrum

**Figure 4:**
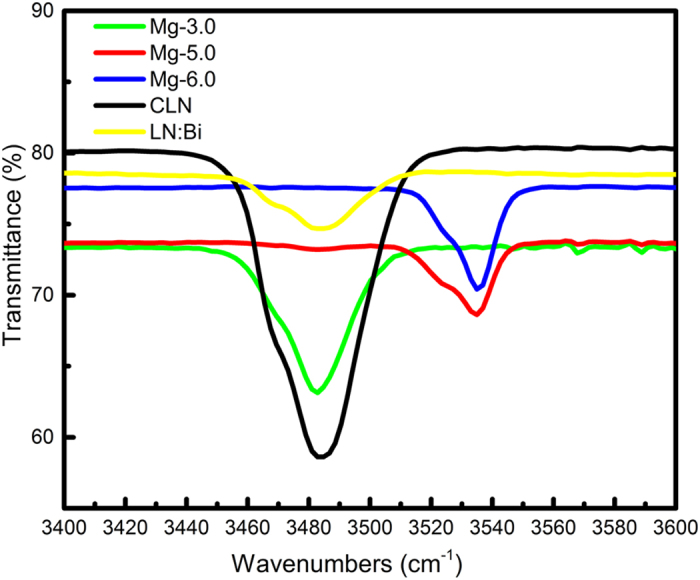
OH^−^ absorption spectra of CLN and LN:Bi,Mg crystals. For comparison, the curves of CLN and LN:Bi were also drawn.

OH^−^ absorption spectrum can also be used to probe the defect structure of LN crystals because OH^−^ stretching vibration is sensitive to the change of ion environment^23^,^24^. As shown in Fig. 4, the OH^−^ absorption peak position of LN:Bi,Mg is different with various Mg doping concentration, it appears at 3484 cm^−1^ for CLN, LN:Bi and LN:Bi,Mg_3.0,_ but at 3535 cm^−1^ for LN:Bi,Mg_5.0_ and LN:Bi,Mg_6.0_. It was reported that the OH^−^ absorption peak appears at about 3484 cm^−1^ for CLN and at about 3535 cm^−1^ for LN:Mg when the doping concentration of Mg exceeds the optical damage resistance threshold^25^,^26^. The above experimental results indicate that doping concentration of Mg in LN:Bi,Mg_3.0_ is below the damage resistance threshold, and that in LN:Bi,Mg_5.0_ and LN:Bi,Mg_6.0_ are above the threshold, which are consistent with the results of optical damage resistance shown in Fig. 2.

### Discussion

The UV-visible absorption spectra of LN:Bi,Mg crystals imply that the dopants may introduce new photorefraction centers into crystals., and the experimental results of Fig. 1 indicate that LN:Bi,Mg crystals are promising photorefractive material, which have very short photorefraction response time and high photorefractive sensitivity. On the other hand, the OH^−^ absorption spectra illustrate that their doping concentration are above the doping threshold of Mg, and the results in Fig. 2 indicate that LN:Bi,Mg_5.0_ and LN:Bi,Mg_6.0_ have strong optical damage resistance. The simultaneous enhancement of photorefraction and optical damage resistance is conflict with the literature. As pointed out in the introduction that optical damage was renamed as photorefraction, that is to say, optical damage and photorefraction is the same thing, which has been proved by plenty of experimental results in the past half century. Then what has happened in LN:Bi,Mg crystals?

At first, we analyzed the mechanism of optical damage and photorefraction. As we known that drift, diffusion, and photovoltaic effect were discovered as the possible reasons for photorefraction^27^,^28^,^29^,^30^. When the crystal is under the action of external electric field, the charge carriers will drift and induce space charge field and photorefraction according to electro-optic effect, but no external electric field was applied in our experiments, so drift needn’t to be considered in this phenomenon. The photovoltaic effect will induce the directional movement of carriers along c-axis, which surely causes enlargement and distortion of the transmitted light beam under a low light intensity. As shown in Fig. 2, the beam shapes transmitted LN:Bi,Mg_5.0_ and LN:Bi,Mg_6.0_ have no apparent change as compared with the original one. Therefore, photovoltaic effect should not dominate the photorefractive process in these crystals. It was reported that optical scattering is more connect with photovoltaic effects than drift and diffusion^31^, so the transmitted beam shapes should distort if photovoltaic effect dominates photorefractive effects. Thus, we can deduce that diffusion should be the dominant reason of the photorefraction of LN:Bi,Mg_5.0_ and LN:Bi,Mg_6.0_, that is also to say that diffusion has little influence on the optical damage of crystals.

**Figure 5:**
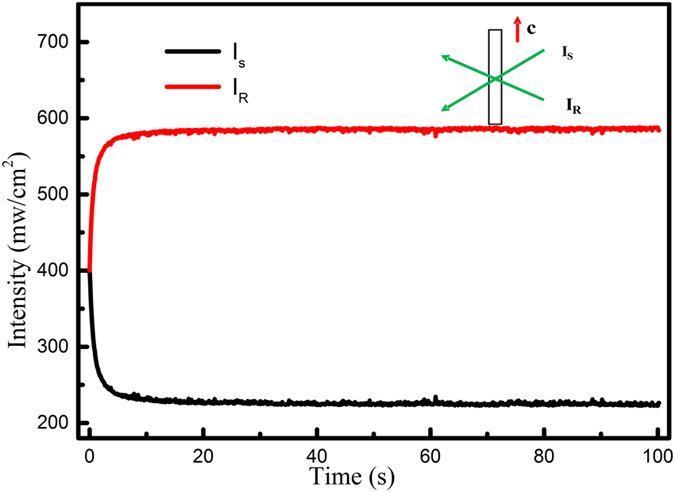
The light energy transfer during the two coupling process of LN:Bi,Mg_6.0_ crystal in 532 nm.

It was reported when the coupled laser beams with equal light intensity transmit LiNbO_3_ y-plate, if the light energy unidirectional transferred towards to +c or −c axis, the dominant charge carriers are holes or electrons, respectively, and the dominant charge transport mechanism is diffusion^32^. So we did this experiment and the typical result was shown in Fig. 5. We can see that the light energy was unidirectional transferred from the I_R_ beam to the I_S_ beam, and the light intensity of two beams keep stable without apparent fluctuation. According this result, we can deduce that diffusion is the dominant charge transport mechanism in the photorefractive process of LN:Bi,Mg_5.0_ and LN:Bi,Mg_6.0_ crystals.

Therefore, we can conclude that diffusion is the dominant mechanism in the photorefraction of LN:Bi,Mg crystals, and the codoping of Bi and Mg may enhance diffusion and weaken the photovoltaic effect in LiNbO_3_ crystals. Our results indicate that diffusion will cause photorefraction but may sight optical damage, photorefraction doesn’t equal to optical damage, optical damage is only an expression of photorefraction, may optically induced inhomogeneity is more suitable for photorefraction than optical damage.

## Conclusions

In a summary, series of LN:Bi,Mg crystals with high optical quality were grown by Czochralski method. The photorefractive response time of LN:Bi,Mg_6.0_ was greatly shortened to 170 ms with a diffraction efficiency of 18% at 488 nm, and the photorefractive sensitivity reached 21 cm^2^/J, which is three orders of magnitude higher than that of CLN. Moreover, the light intensity that LN:Bi,Mg_5.0_ and LN:Bi,Mg_6.0_ can withstand without optical damage is above 10^6^ W/cm^2^. Our experimental results indicate that the co-doping of Bi and Mg can enhance not only the photorefractive properties but also the optical damage resistance, which is very important for the practical applications of volume holographic storage, integrated optic application and 3D display. Diffusion is the dominant mechanism in the photorefractive process of LN:Bi,Mg crystals, it seems that optical damage is sight when diffusion dominates the photorefractive process, which means photorefraction doesn’t equal to optical damage.

## Methods

### Samples preparation

A series of congruent LN crystals doped with 1.0 mol% Bi and co-doped with different concentration of Mg were grown along the z-axis with the conventional Czochralski method. The congruent composition was selected as [Li]/[Nb] = 48.38/51.62, and the concentrations of MgO were 3.0 mol%, 5.0 mol% and 6.0 mol%, labeled as LN:Bi,Mg_3.0_, LN:Bi,Mg_5.0_ and LN:Bi,Mg_6.0_. The as-grown LN:Bi,Mg crystals were about 35 mm along z-axis with a diameter of 30 mm. After the crystal growth process, the as-grown crystals were annealed and polarized in a furnace with uniform temperature, and a DC current with the density of 5 mA/cm^2^ was given to the crystals after the temperature of furnace had been keeping at 1190 °C for 20 h. Then 3.0 mm and 1.0 mm-thick plates along y-face were cut and polished to optical grade for properties measurements. The dimensional sizes of these plates along z and x directions were 30 × 20 mm^2^. For comparison, CLN and 1.0 mol% Bi mono-doped LN (LN:Bi) crystals were also grown and sliced.

### Optical spectrum measurements

The UV-visible absorption spectra of LN:Bi,Mg crystals were measured at room temperature by an UV-4100 spectrophotometer with light transmitting through the 1.0 mm thick y-plates, and the IR absorption spectra of LN:Bi,Mg crystals were measured by a FTIR spectrometer with the incident light perpendicular to the y plates. The energy accuracy of the spectrometer is better than ±0.5 cm^−1^.

## Additional Information

**How to cite this article**: Zheng, D. *et al*. The simultaneous enhancement of photorefraction and optical damage resistance in MgO and Bi_2_O_3_ co-doped LiNbO_3_ crystals. *Sci. Rep*. **6**, 20308; doi: 10.1038/srep20308 (2016).
